# When perception is reality: Resident perception of faculty gender parity in a university-based internal medicine residency program

**DOI:** 10.1007/s40037-019-00532-9

**Published:** 2019-11-14

**Authors:** Jennifer Reilly Lukela, Aditi Ramakrishnan, Nicole Hadeed, John Del Valle

**Affiliations:** 1 Department of Internal Medicine, University of Michigan, Ann Arbor, MI USA; 2 Division of Infectious Diseases, Emory University School of Medicine, Atlanta, GA USA

**Keywords:** Medical education-graduate, Gender disparity, Implicit bias, Role model

## Abstract

**Introduction:**

Although women have entered medical school and internal medicine residency programs in significant numbers for decades, women faculty remain underrepresented in senior and departmental leadership roles. How residents perceive this gender disparity is unknown. We sought to assess resident perception of gender parity among departmental leadership and teaching faculty in our internal medicine department, and to determine the actual gender distribution of those faculty roles.

**Methods:**

An anonymous cross-sectional survey was distributed to evaluate resident perception of gender representation of various faculty roles. Using conference schedules, resident evaluations, and our department website, we determined the actual representation of women faculty in department leadership roles, and in clinical and educational activities.

**Results:**

88 of 164 residents (54%) responded. Women residents were less likely than men to perceive that women faculty were equally represented in department leadership (45% men agreed vs. 13% women, *p* < 0.05), clinical teaching roles (55% men agreed vs. 28% women, *p* < 0.05), or facilitating educational conferences (45% men agreed vs. 28% women, *p* = 0.074). In 2017, the internal medicine department at our institution comprised 815 faculty members, 473 men (58%) and 342 women (42%). At that time, women faculty held 5% of senior departmental leadership positions and 21% of educational leadership positions. During the year preceding survey distribution, women faculty attended on internal medicine inpatient wards for 33% of the total number of weeks, staffed 20% of morning reports, and facilitated 28% of noon conferences.

**Discussion:**

Women residents in our internal medicine training program perceived a gender disparity among faculty in leadership and educational positions to a greater extent than male residents. The perception of women trainees was accurate. In addition to disproportionate underrepresentation in leadership positions, women faculty were underrepresented in prominent educational positions, including attending on inpatient services and serving as discussants at educational conferences.

**Electronic supplementary material:**

The online version of this article (10.1007/s40037-019-00532-9) contains supplementary material, which is available to authorized users.

## What this paper adds

Disparities in the representation of women faculty in senior and leadership positions in academic medicine are well described. The impact of this disparity on trainees, particularly women trainees, in graduate medical education is unknown. We sought to understand resident perception of gender parity among the faculty within our internal medicine residency program. We compared resident perception with *actual* faculty gender representation. Our results suggest that women residents do recognize faculty gender disparities and that women residents perceive these disparities to a greater extent than their male colleagues. The lack of gender-concordant role models in educationally prominent and senior leadership positions has the potential to perpetuate continued gender disparities in academic medicine.

## Introduction

Women have represented greater than 40% of US medical school graduates for more than two decades, and in 2017 women matriculates at US medical schools outnumbered men for the first time [1, 2]. The percentage of women entering internal medicine residency programs has also been stable at 43–46% over the past decade [3, 4]. Despite the relative gender parity of those entering the field of internal medicine, a significant disparity has persisted in the number of women faculty in senior and leadership positions in departments of internal medicine, part of a phenomenon often referred to as ‘the leaky pipeline.’ In 2015, only 21% of full professors in internal medicine and 13% of internal medicine department chairs were women [5–7]. It has been postulated that the paucity of women physicians in prominent positions may perpetuate the leaky pipeline due to a lack of role models for women trainees. Few studies have been conducted, however, to assess whether women trainees actually notice these gender disparities in positions of leadership or educational prominence.

In the present study, we sought to better understand the gender climate within our own internal medicine residency program. Specifically, we wanted to understand the perception among both male and female residents regarding parity of gender representation in our department leadership and distinguished academic roles. In addition to assessing resident *perception* of gender parity, we sought to understand the *actual* level of gender parity in leadership roles, both administrative and educational, within our department and our residency program. Specifically, we assessed for gender equity among faculty in ‘high-contact’ educational roles. We defined faculty as ‘high-contact’ if they attended on the inpatient wards, staffed outpatient resident continuity clinics, or if they served as facilitators of the morning report and other educational resident conferences.

## Methods

In December 2017, we conducted a cross-sectional survey of residents in our training program to assess the gender climate. At that time, the University of Michigan Internal Medicine Residency Program consisted of 130 residents (49 women and 81 men) with 4 chief medical residents (2 women and 2 men). In addition, 32 residents (24 women and 8 men) from the University of Michigan Medicine and Paediatrics Residency Program rotated on our teaching services and participated in our educational programming. At the time this survey was conducted, no openly identified non-binary individuals were completing their training in our residency program. Our residents rotate on clinical services both at the University Hospital, a 550-bed tertiary care facility, and the Ann Arbor Veterans Affairs Hospital, a 102-bed referral hospital for specialty care.

The survey was developed in October 2017. Initial survey design and content was informed by a literature review. Survey content and format was further refined using input from the directors of our internal medicine and medicine and paediatrics residency programs and with guidance from the director for Research and Evaluation at the University of Michigan ADVANCE program, which had conducted similar gender climate assessment surveys on a university-wide level in the past. Both men and women residents received the same gender climate survey but the survey sent to women residents also included a needs assessment for the purpose of curriculum development (see the online Electronic Supplementary Material for the survey). The survey was initially pilot tested on three residents to assess appropriateness of survey length and ease of completion. The survey was distributed electronically to all residents in the internal medicine and medicine and paediatrics programs in December 2017. The chief medical residents were not included in the survey population. Three email reminders were sent to encourage survey completion (two from resident colleagues and one from an associate program director).

For survey response analysis, items with 5‑point Likert scale ‘strongly agreed’ and ‘agreed’ responses and ‘disagree’ and ‘strongly disagree’ responses were combined. Responses between men and women respondents were compared using the Chi-squared test. Data regarding gender distribution of faculty speakers and facilitators at educational conferences were obtained via review of the conference schedule for the calendar year 2017 at both the University and Veterans Affairs Hospitals. Data regarding the demographics of faculty attending on clinical teaching services were collected using faculty evaluations completed by residents on the various clinical teaching services and in continuity clinics during that year. Data regarding the gender balance of department, divisional and education leadership roles (i.e., residency and fellowship program directors) were obtained through review of our department website in December 2017. Parity of gender distribution of faculty teaching roles, relative to gender distribution on the entire internal medicine faculty, was compared using the Z score test for two proportions. Parity of gender distribution for the leadership roles was analysed using both the Chi-squared test and Fisher’s exact test due to small sample sizes in some leadership categories.

This study was reviewed by the University of Michigan Medical School Institutional Review Board and deemed exempt. All procedures performed in studies involving human participants were in accordance with the ethical standards of the institutional and/or national research committee and with the 1964 Helsinki Declaration and its later amendments or comparable ethical standards.

## Results

A total of 88 residents, or 54% of the total residents surveyed, completed the survey, with 63% (46 of 73) of women and 47% (42 of 89) of men responding. Regardless of gender, the majority of residents in our training programs, reported being highly satisfied with their experience in residency training (Tab. 1). While 45% of male residents agreed that women were appropriately represented in senior positions in the department of internal medicine, only 13% of women respondents agreed with this statement (*p* = 0.000773). Similarly, while 55% of male residents agreed that there was equal representation of men and women faculty attending on the inpatient and outpatient teaching services, only 28% of women residents agreed that there was gender parity among teaching faculty (*p* = 0.006194). More men than women residents also felt that there was gender parity regarding faculty leading educational conferences such as morning report and grand rounds (45% of men agreed vs. 28% of women), though this difference was not statistically significant (*p* = 0.0741).

**Table 1:** Resident perception of gender parity

	Men residents		Women residents		*P* value
	*Extremely unsatisfied/unsatisfied*^*a*^	*Satisfied/extremely satisfied*^*a*^	*Extremely unsatisfied/unsatisfied*^*a*^	*Satisfied/extremely satisfied*^*a*^	*–*
Are you satisfied with your residency training experience?	0 (0%)	34 (94%)	0 (0%)	32 (97%)	N/A
	*Strongly disagree/disagree*^*a*^	*Agree/strongly agree*^*a*^	*Strongly disagree/disagree*^*a*^	*Agree/strongly agree*^*a*^	*–*
Women are appropriately represented in senior positions in the department of internal medicine	17 (41%)	19 (45%)	32 (70%)	6 (13%)	*p* = 0.000773
There is equal representation of both men and women teaching faculty on the wards and in clinics	11 (26%)	23 (55%)	24 (52%)	13 (28%)	*p* = 0.006194
There is equal representation of both men and women faculty as discussants at educational conferences and grand rounds	14 (33%)	19 (45%)	23 (50%)	13 (28%)	*p* = 0.0741

^a^Based on 5‑point Likert scale

**Fig. 1:**
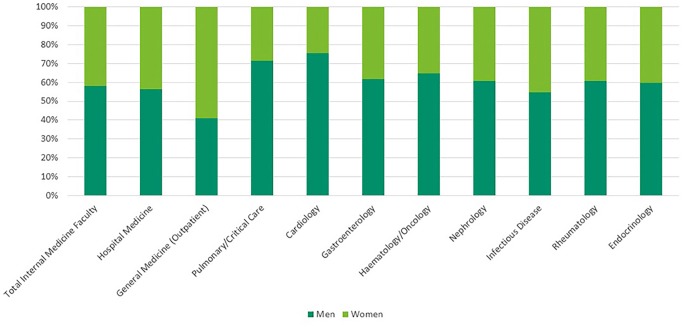
Faculty gender distribution by division, Department of Internal Medicine (2017)

**Fig. 2:**
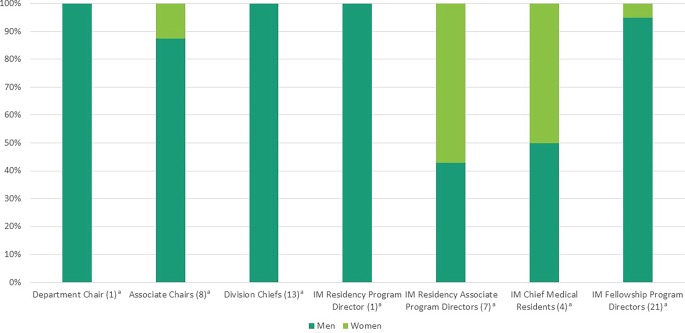
Gender distribution of leadership roles, Department of Internal Medicine (2017) (^a^total number of positions available) [23–25]

At the time of survey distribution, our internal medicine department comprised 815 faculty members, 473 men (58%) and 342 women (42%) (Fig. 1). During that year, male faculty had an average total full-time equivalent (FTE) of 0.985, while women had an average FTE of 0.943. In 2017, the Chair of the Department of Medicine at the University of Michigan was a man and 13 of 13 division chiefs (100%) were men. While there was greater gender parity among the leadership of the internal medicine residency program, 91% of internal medicine fellowship program directors were men (Fig. 2). When the total number of women faculty in leadership positions, both departmental and educational, was compared with the total number of women on the internal medicine faculty, women were found to be significantly underrepresented in these roles relative to their proportion on the faculty (Tab. 2).

**Table 2:** Gender distribution and relative gender parity in leadership and faculty teaching roles, Department of Internal Medicine (2017)

	Men	Women	*P* value
Departmental leadership roles^a^	21 (95%)	1 (5%)	*p* = 0.000312 (Chi-squared)^b^ *p* = 0.0001 (Fisher exact)^b^
Educational leadership roles^c^	23 (79%)	6 (21%)	*p* = 0.018086 (Chi-squared)^b^ *p* = 0.0206 (Fisher exact)^b^
Total inpatient faculty attending weeks	1298 (69%)	578 (31%)	*p* < 0.00001 (Z score test for two proportions)^b^
Total intensive care unit attending weeks	260 (84%)	50 (16%)	*p* *=0.0271 *(Z score test for two proportions)^d^
Continuity clinic attending	23 (45%)	28 (55%)	*p* = 0.62414 (Z score test for two proportions)^e^
Inpatient morning report facilitators	172 (80%)	42 (20%)	*p* < 0.00001 (Z score test for two proportions)^b^
Ambulatory morning report facilitators	142 (69%)	63 (31%)	*p* = 0.00398 (Z score test for two proportions)^b^
Noon conference facilitators	42 (72%)	16 (28%)	*p* *=0.03662 *(Z score test for two proportions)^b^

^a^Pooled department chair, associate chairs and division chiefs

^b^Compared with gender representation in overall internal medicine faculty

^c^Pooled program director, associate program directors, and fellowship program directors

^d^Compared with gender representation in pulmonary and critical care faculty only

^e^Compared with gender representation in division of general medicine only

**Fig. 3:**
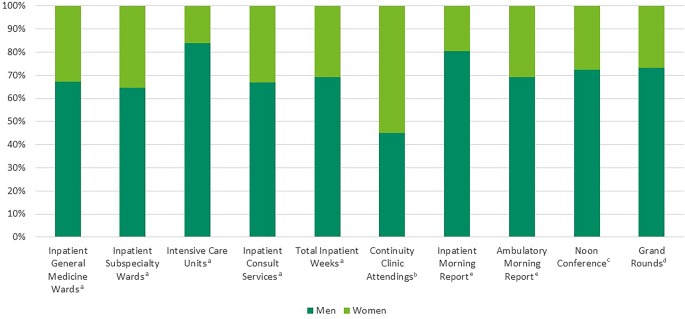
Gender distribution of teaching faculty on clinical services or education conference facilitator/presenter, Department of Internal Medicine (2017) (^a^inpatient time measured in number of weeks per year, ^b^continuity clinic attendings assigned to half day per week for whole academic year, ^c^occurs daily Monday–Friday, ^d^occurs two times weekly, ^e^occurs once weekly (excluded monthly Morbidity and Mortality conference lead by CMRs from analysis))

During the 2017 calendar year (the year preceding survey distribution), 69% of the total weeks on inpatient clinical services were staffed by male faculty, while 31% of the weeks were staffed by female faculty (Fig. 3). When the total number of weeks on inpatient teaching services (including inpatient wards, ICUs, inpatient consult services) staffed by women faculty was compared with the total number of women faculty in the department of internal medicine, women were significantly underrepresented compared with their male colleagues (Tab. 2). There was variation of gender parity of faculty representation based on inpatient service type, with only 16% of inpatient weeks in the intensive care units being staffed by women faculty, while 35% of weeks on subspecialty services (i.e., haematology-oncology, gastroenterology-hepatology, cardiology) and 33% of weeks on general medicine ward services were staffed by women. Some of this service-specific variation was likely due to differences in the number of women on the specialty division faculty. For example, only 29% of faculty within the Division of Pulmonary and Critical Care were women, while 44% of the Division of Hospital Medicine faculty were women. However, the proportion of women staffing in our ICUs was still lower than would be expected based on their relative proportion on the pulmonary and critical care medicine faculty (Tab. 2). Outpatient general medicine continuity clinics represented the only clinical service that was primarily staffed by female faculty, with 55% of continuity clinic attending roles being filled by women. This was concordant with the fact that the Division of General Medicine was the only division with a majority of women faculty (59%) (Fig. 1).

Only 20% of inpatient morning report faculty discussants were women and only 28% of noon conference speakers were women faculty. Ambulatory morning report, the residency conference focused on outpatient general medicine, was also facilitated predominately by male faculty (69% men vs. 31% women faculty facilitators) (Fig. 3). The gender disparity of facilitators for each of these conferences was significant relative to the proportion of women on the faculty (Tab. 2). During the 2017 calendar year, 27% of invited internal medicine grand rounds speakers at our institution were women.

## Discussion

Women residents in our internal medicine training program perceived a gender difference in the representation of women faculty in positions of senior leadership, as well as in positions of educational prominence within our department. After review of departmental leadership data, attending and conference schedules, we confirmed that the perception of our women trainees that a gender disparity existed in these domains was a reality. Women faculty within the Department of Internal Medicine at our institution did have a significantly lower representation in positions of leadership and educational prominence, disproportionate to their overall gender representation on our faculty.

Though women have represented nearly half of all medical school graduates for more than two decades, women continue to leave academic medicine at a higher rate than their male peers and lag behind in terms of promotion and positions of leadership [1–7]. Many factors have been postulated to play a causal role in the persistence of the leaky pipeline for women faculty in academic medicine. Prior work has addressed the potential role of the disproportionate burden of family and domestic responsibilities on women compared with their male counterparts [8–10]. Some have suggested that women may be more likely to work ‘part time’ and that this may be a cause for slower professional advancement and inclusion in leadership positions. While this may be a factor in some cases, for our internal medicine faculty the average total FTE for men and women faculty was not markedly different.

Issues of unequal compensation and disparate distribution of grants and funding based on gender persist [11–13]. Additionally, there is evidence that women continue to be promoted at a lower rate despite equal qualifications and years of experience, as well as research and teaching accomplishments [5]. Women faculty have been reported to receive less encouragement to pursue promotion or advancement, to have less access to ‘informal networks’ that facilitate career advancement, and to perceive that they will have less ‘personal benefit’ from academic advancement compared with their male peers [14, 15]. Lack of awareness of gender disparities and unconscious or implicit bias have also been cited as possible factors in the persistent gender climate and underrepresentation of women physicians in top leadership positions in academic medicine [16, 17]. Implicit bias, in particular, has been thought to play a central role in the slower advancement of women in a variety of professions and fields, with numerous examples revealing that identical work is rated lower by both men and women evaluators when it is believed to have been completed by a woman [18, 19].

Another potential contributor to the leaky pipeline is the lack of gender concordant role models for younger women trainees and female junior faculty in academic medicine. Though previous studies have revealed inequity of faculty representation based on gender at premier academic medicine conferences such as at grand rounds [20], our study also demonstrated gender disparities for faculty roles in the conferences more regularly attended by trainees, including residents, interns and medical students rotating on clinical services, such as the morning report. Our study is also among the first to directly assess whether trainees recognize this gender disparity among faculty roles, and if the perception of trainees is different based on their own gender. In addition to adding credence to the theory that this lack of role models may play a significant role in the mindset of our women trainees, we believe that the faculty gender disparities uncovered by our analysis also provides a potential ‘fixable problem.’ At our own residency program, in response to the data discovered through this study, we are taking active steps to address the gender disparity of faculty leading and facilitating our academic conferences. In addition to reviewing our list of ‘invited faculty’ to facilitate our educational conferences to improve gender diversity, we have begun actively monitoring the gender of conference facilitators. We review these numbers periodically throughout the year to ensure more equal representation of both men and women faculty discussants.

Interestingly, the degree to which women trainees appreciated these gender disparities was greater than that of their male colleagues. We suspect that this is yet another manifestation of unconscious or implicit bias. Similar differences in perception of the gender climate between men and women have been observed in the business world. This was demonstrated in the 2017 *Women in the Workplace* report by LeanIn.Org and McKinsey & Company, where there were striking differences in perception of workplace gender climate between men and women employees included in the survey. As noted by the authors, ‘(o)ne of the most powerful reasons for the lack of progress is a simple one: we have blind spots when it comes to diversity, and we can’t solve problems that we don’t see or understand clearly ‘ [21].

Our study is limited as it represents the experience of only one graduate medical education program at a single institution. However, our grand rounds data were congruent with previously reported national data and other internal medicine residency programs have also reported similar gender disparities that have existed in more informal recognition programs of resident research and scholarship endeavours [20, 22]. Therefore, we suspect that our experience may not be different from that of our peer departments or peer institutions. Our study may also be limited by a modest survey response rate with over-representation of women residents in our program compared with male residents. This phenomenon of a disparate gender response rate is in itself interesting and may be a reflection of the previously reported trend that women in academic medicine are more likely to engage in ‘collegial’ activities or activities less likely to result in measurable ‘academic productivity ’ [14]. Alternatively, that difference may reflect an inherent gender difference in the overall interest in the issue of gender disparities as the topic of the survey (‘assessment of the gender climate’) was included in the soliciting email. Similarly, it is possible that inclusion of the needs assessment on the survey sent to women residents may have ‘primed’ women residents to be more aware of gender disparities. However, the needs assessment portion of the survey sent to women was intentionally placed after the gender climate survey questions to avoid introducing this potential source of bias.

Despite these limitations, we believe that this work adds to the growing literature regarding persistent gender disparities in medicine, including within the world of graduate medical education. With increased attention to the potential importance to women trainees of frequent contact with same-sex role models in prominent academic positions, we believe we can continue to advance efforts toward increased gender parity in educational and leadership roles in academic medicine. Through such efforts, along with interventions to support women faculty with work-life balance, greater mentorship and sponsorship, and more equity in compensation and funding, perhaps we can begin to meaningfully fix the leaky pipeline.

## Caption Electronic Supplementary Material

Gender Climate Survey and Needs Assessment for Women in Medicine Curriculum
