# Supplementary material for: When perception is reality: Resident perception of faculty gender parity in a university-based internal medicine residency program
Source: Perspect Med Educ. 2019 Nov 14;8(6):346–52. doi: 10.1007/s40037-019-00532-9 (PMC6904409; doi:10.1007/s40037-019-00532-9)
Supplement: Supplementary file 1 — Gender Climate Survey and Needs Assessment for Women in Medicine Curriculum [file 40037_2019_532_MOESM1_ESM.docx]

**Gender Climate Survey**

|  | Question | Strongly Agree | Agree | Neutral | Disagree | Strongly Disagree |
| --- | --- | --- | --- | --- | --- | --- |
| 1 | Gender bias/discrimination is a problem in the University of Michigan Internal Medicine Residency Program |  |  |  |  |  |
| 2 | Female/Male residents in Internal Medicine are well supported at Michigan Medicine |  |  |  |  |  |
| 3 | Faculty staffing residents on the wards or in clinic treat men and women residents the same |  |  |  |  |  |
| 4 | In residency program meetings/conferences, people pay equal attention when women speak as when men do |  |  |  |  |  |
| 5 | I have witnessed sexist remarks toward trainees in the clinical environment |  |  |  |  |  |
| 6 | I have witnessed sexist remarks toward trainees in educational conferences (ie, morning report, grand rounds) |  |  |  |  |  |
| 7 | Women are appropriately represented in senior positions within the Department of Internal Medicine |  |  |  |  |  |
| 8 | There is equal representation of both men and women as teaching faculty on the wards/in clinics |  |  |  |  |  |
| 9 | There is equal representation of both men and women faculty as discussants during educational conferences/grand rounds |  |  |  |  |  |
| 10 | There is equal access for both men and women residents to find mentors for research projects |  |  |  |  |  |

|  | Question | Never | 1-2 times over the course of training | Several times over course of training | 1-2 times per month | At least weekly |
| --- | --- | --- | --- | --- | --- | --- |
| 11 | How often have you experienced gender bias/discrimination during your training at Michigan Medicine? |  |  |  |  |  |
| 12 | How often have you witnessed gender bias/discrimination during your training at Michigan Medicine? |  |  |  |  |  |

13. In what setting have you experienced or witnessed gender bias/discrimination? (please select all that apply)

a. Inpatient wards as University Hospital

b. VA inpatient wards

c. Intensive care units at University Hospital (CCMU/CCU)

d. VA MICU

e. Outpatient clinics

f. Educational conferences (ie, morning report)

g. Prefer not to say

14. Who demonstrated the gender bias/discrimination? (please select all that apply)

a. Patients

b. Ancillary/support staff (nurses, physical therapists, respiratory therapists)

c. Peers/colleagues

d. Faculty

e. Program or Department administration

f. Prefer not to say

15. I feel well prepared to respond to gender bias/discrimination when I encounter it.

a. Strongly agree

b. Agree

c. Neutral

d. Disagree

e. Strongly disagree

16. Who do you identify as a potential source of support in the event that you encounter gender bias/discrimination?

a. Faculty within the Department of Internal Medicine (outside of continuity clinic faculty)

b. Continuity clinic faculty

c. Colleagues

d. Program directors

e. Family/peers outside of residency program

f. Other

g. I do not have an identified source of support to turn to in the event of gender discrimination

17. Are you a part of the Internal Medicine or Medicine-Pediatrics residency program?

a. Internal Medicine

b. Medicine- Pediatrics

18. What is your PGY level?

a. HO1

b. HO2

c. HO3

d. HO4

|  | Question | Extremely Satisfied | Satisfied | Neutral | Unsatisfied | Extremely Unsatisfied |
| --- | --- | --- | --- | --- | --- | --- |
| 19 | Are you satisfied with your residency training experience at the University of Michigan? |  |  |  |  |  |
| 20 | Are you satisfied with your work-life balance |  |  |  |  |  |

21. Do you have children?

a. Yes

b. No

22. Do you have someone that you identify as a mentor or sponsor?

a. Yes

b. No

**Needs Assessment (given to women residents only)**

1. Which of the following would you find helpful in terms of your career development and/or addressing gender issues in medicine? (select all that apply)
   1. Mentor/coach
   2. Women in Medicine seminar/workshop series
   3. Networking events regarding clinical and research interests
   4. Awards for research and conference funding pertaining to gender issues in medicine
2. Which of the following topics would you like to see addressed in a program within the residency program for women in medicine? (please select top four)
   1. Work-Life Balance
   2. Leadership Skills
   3. Negotiation strategies/crucial conversations
   4. Career advancement skill development
   5. Strategies to counter gender bias in the workplace
   6. Overcoming imposter syndrome/executive presence
   7. Mentorship and sponsorship
   8. Other (free text write in)
